# Urticarial hypocomplementemic vasculitis syndrome and systemic lupus erythematosus: a case report and review of the literature

**DOI:** 10.3389/fimmu.2025.1649699

**Published:** 2025-08-07

**Authors:** Yasmina Ouerdani, Tayssir Ben Achour, Ahlem Ben Hmid, Fatma Said, Imen Ayadi, Alia Zehani Kassar, Maysam Jridi, Imed Ben Ghorbel, Ines Naceur, Samar Samoud, Yousr Galai, Lilia Laadhar, Slim Haouet, Maryam Kallel Sellami, Monia Smiti, Imen Zamali, Mélika Ben Ahmed

**Affiliations:** ^1^ Clinical Immunology Department, Pasteur Institute of Tunis, Tunis, Tunisia; ^2^ Faculty of Medicine of Tunis, University of Tunis El Manar, Tunis, Tunisia; ^3^ Department of Internal Medicine, Rabta University Hospital, Tunis, Tunisia; ^4^ Laboratory of Transmission, Control and Immunobiology of Infection, Pasteur Institute of Tunis, Tunis, Tunisia; ^5^ Immunology Laboratory, Rabta University Hospital, Tunis, Tunisia; ^6^ Department of Pathology, Rabta University Hospital, Tunis, Tunisia

**Keywords:** hypocomplementemic urticarial vasculitis syndrome, systemic lupus erythematosus, case report, autoimmune disease, overlap syndrome

## Abstract

**Background:**

Hypocomplementemic urticarial vasculitis (HUV) syndrome is a rare form of small-vessel vasculitis characterized by a heterogeneous spectrum of clinical and biological findings. It is typically marked by chronic urticarial eruptions, hypocomplementemia and histopathological evidence of leukocytoclastic vasculitis. It may also involve multiple organ systems, with frequent articular, gastrointestinal, renal, and other systemic manifestations. The differential diagnosis with other systemic autoimmune diseases, particularly systemic lupus erythematosus (SLE), is often challenging due to their frequent association and the blurred boundaries between these entities.

**Case presentation:**

We report the case of a 34-year-old Tunisian man with an association of HUV and SLE. The diagnosis of SLE was established according to the 2019 European League Against Rheumatism (EULAR) criteria, based on the combination of inflammatory polyarthralgia, lymphopenia, a high titer of anti-nuclear antibodies, specific anti-Sm and anti-DNA antibodies, and consumption of C3 and C4 complement fractions. The diagnosis of HUV was made based on the presence of two major criteria: chronic urticaria and hypocomplementemia, along with four minor criteria: leukocytoclastic vasculitis, recurrent abdominal pain, episcleritis, and the presence of anti- C1q antibodies.

**Conclusion:**

HUV and SLE share key clinical, immunological, and pathophysiological features, suggesting that they may lie along the same spectrum of autoimmune diseases. Their association, as seen in our patient, has been described in the literature. This overlap may result in more severe disease and requires close clinical follow-up.

## Introduction

Hypocomplementemic urticarial vasculitis (HUV) syndrome, or McDuffie syndrome, is a rare autoimmune disorder first described in 1973 (1). It typically affects individuals in the fourth to fifth decades of life and exhibits a marked female predominance, with a ratio of approximately 8:1 (2). The true incidence of HUV is difficult to ascertain due to its wide range of clinical manifestations, diagnostic challenges, and overall rarity, but it is estimated to be around 0.5 cases per 100,000 individuals (3). Although traditionally regarded as a distinct clinical entity, increasing evidence highlights its overlap with systemic lupus erythematosus (SLE) (4, 5). Both HUV and SLE are autoimmune diseases that share a spectrum of clinical and immunological features, including arthralgia, cutaneous involvement, and hypocomplementemia. However, they differ in underlying pathophysiology, prognosis, and therapeutic approaches. The polymorphic nature of HUV, which may occasionally include features typically associated with SLE, challenges the distinction between these two conditions. This overlap has prompted debate among researchers over whether HUV represents a distinct entity or falls within the broader spectrum of lupus-related disorders (6).

Accurate differentiation between HUV and SLE is crucial, as it influences diagnosis, treatment strategies, and long-term prognosis. Here, we present the case of a 34-year-old Tunisian man with overlapping features of both conditions. This rare association exemplifies the diagnostic and therapeutic challenges encountered in such cases and underscores the importance of comprehensive clinical assessment and targeted immunological evaluation.

## Case report

**Figure 1:**
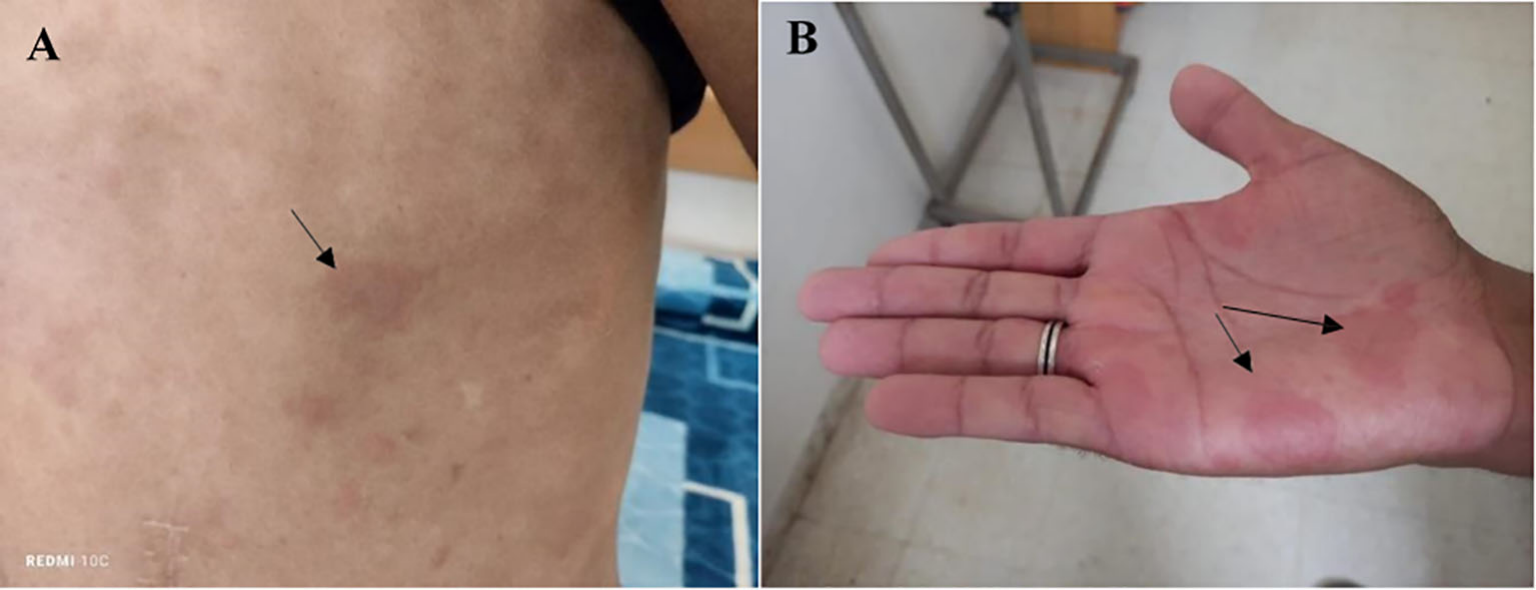
Papular, erythematous, and edematous cutaneous lesions exhibiting an annular distribution over the trunk **(A)** and palmar surfaces **(B)**, clinically consistent with urticarial eruptions.

A 34-year-old Tunisian patient with a history of appendectomy in March 2023 and recurrent episodes of spontaneously resolving subocclusive syndrome, was referred, in April 2024, to the internal medicine department for further investigation of inflammatory polyarthralgia associated with a skin rash that flared up and spontaneously regressed within 24 to 72 hours. There was no known family history of autoimmune disease. Upon physical examination, the patient was afebrile, had a papular, erythematous, edematous, annular rash on the trunk, neck, and palms, consistent with urticaria (**Figure 1**). The clinical photographs do not fully capture the characteristic features of urticarial vasculitis, given the transient and migratory nature of the eruptions. The eruptions were notably associated with intense pruritus. Abdominal examination was normal. There was a red left eye, but no pain or visual acuity loss. Ophthalmologic examination revealed signs of episcleritis.

Biological tests revealed an inflammatory syndrome with a C-reactive protein (CRP) of 132 mg/l, and serum protein electrophoresis showed hypoalbuminemia (31 g/l), hyper-α1 (4.1 g/l), and polyclonal hypergammaglobulinemia (36.5 g/l). The complete blood count revealed microcytic hypochromic anemia (hemoglobin 9.8 g/dL, MCV 73 fL, reticulocytes 50.000/mm³, ferritin 427 µg/ml) and lymphopenia (930/mm³). Renal function was normal, and 24-hour proteinuria was negative. Hepatitis B and C and HIV viral serologies were negative.

**Figure 2:**
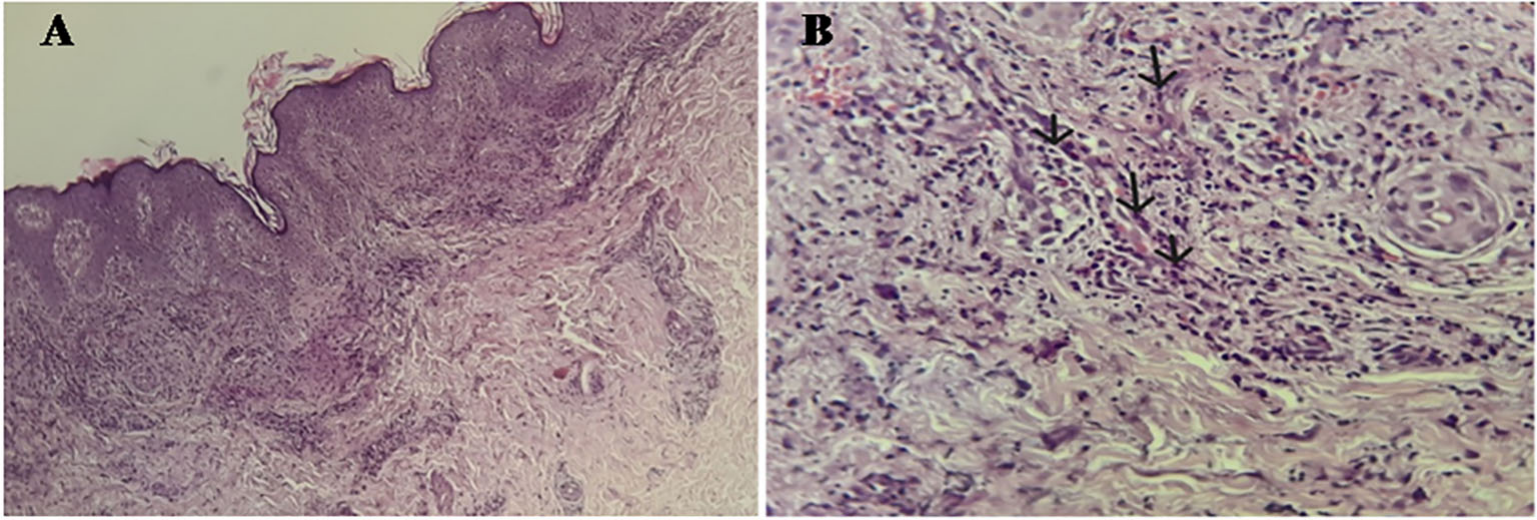
**(A)** Hematoxylin-Eosin x 10: Skin tissue showing a predominantly perivascular inflammatory infiltrate in the dermis without signs of neutrophilic dermatosis. **(B)** Hematoxylin-Eosin x 40: Swelling of the vessel wall with transmural infiltration of neutrophils (arrows), resulting in lesions of leukocytoclastic vasculitis.

On immunological workup, indirect immunofluorescence (IFI) on Hep2 cells revealed a speckled pattern of positive antinuclear antibodies (ANA) with a strong titer of 1/1600. Immunodot testing revealed positivity for anti-Sm (+++), anti-dsDNA (+++), and anti-SSA (++). The presence of anti-dsDNA antibodies was confirmed by IFI on Crithidia luciliae. Complement protein testing showed decreased C3 (0.677 g/L) and C4 (0.111 g/L) fractions, with a normal C1q level (0.199 g/L). The hemolytic complement test (CH50) was reduced to 66%. Anti-C1q antibodies were positive at 49 U/ml by enzyme immunoassay. ANCA testing by IFI was negative. Skin biopsy showed infiltration of the dermis and subcutaneous tissue by neutrophils and lympho-plasmacytes, with exocytosis along the vessel walls, consistent with leukocytoclastic vasculitis. Additionally, nuclear debris around the vessels and within the dermis, as well as extravasation of erythrocytes between collagen fibers and the dermis, were observed (**Figure 2**).

**Figure 3:**
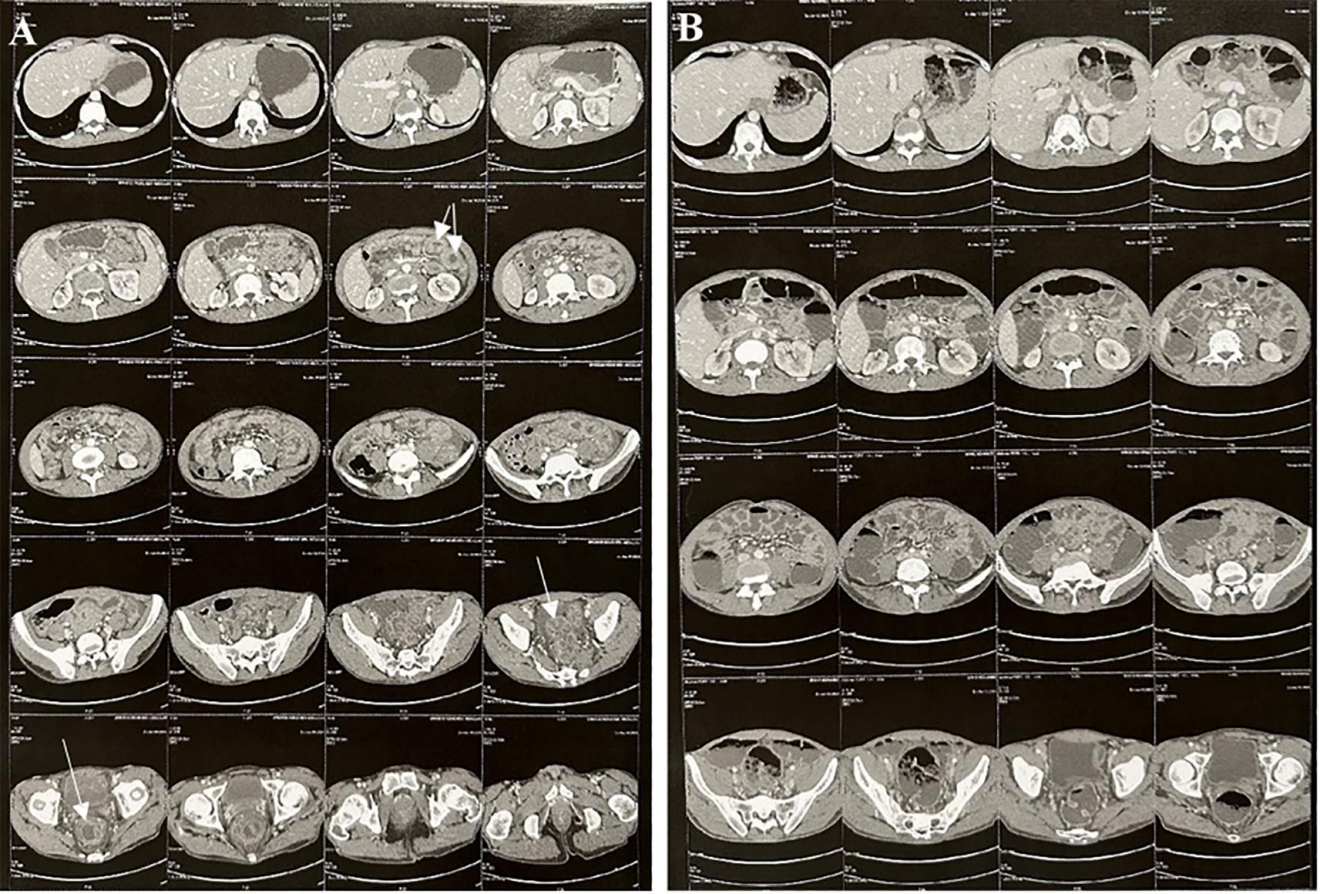
Abdominal and pelvic CT imaging. **(A)** CT scan during the acute phase: Symmetric, uniform thickening of the rectosigmoid and proximal jejunal walls without signs of narrowing, due to submucosal and mucosal edema, accompanied by pronounced mucosal enhancement producing an “accordion sign” appearance. Moderate amount of free fluid in the abdominal cavity. **(B)** Follow-up CT scan: Complete resolution of the previously noted bowel wall thickening in the rectosigmoid and proximal jejunum.

Computed tomography of the abdomen performed during the episode of abdominal pain and subocclusive syndrome revealed rectosigmoid colitis associated with jejunal enteritis and moderate peritoneal effusion. A follow-up CT scan a week later showed complete regression of the inflammatory lesions (**Figure 3**). A chest CT scan was unremarkable.

The diagnosis of SLE was made according to the 2019 EULAR criteria, based on the combination of inflammatory polyarthralgia, lymphopenia, a high-titer ANA, the positivity of specific antibodies like anti-Sm and anti-dsDNA, and consumption of C3 and C4 complement fractions. The diagnosis of HUV was also established based on the presence of two major criteria: chronic urticaria and hypocomplementemia, along with four minor criteria: leukocytoclastic vasculitis, recurrent abdominal pain, episcleritis, and the presence of anti-C1q antibodies.

The patient was initiated on hydroxychloroquine at a dosage of 5 mg/kg/day, resulting in a rapid clinical improvement. Within a few days, urticarial lesions and abdominal pain resolved, and polyarthralgia subsided. Over the 12-month follow-up, the patient remained clinically stable, with no recurrence of symptoms. He experienced no episodes of urticaria, abdominal pain, or lupus flares—particularly renal involvement. Urinalysis consistently showed no proteinuria, and serum creatinine levels remained within normal limits. Immunosuppressive therapy was not required; the patient continued hydroxychloroquine alone, which was well tolerated. This favorable course supports a conservative treatment strategy in selected cases with mild overlapping features and no major organ involvement. However, long-term prognosis remains closely tied to the extent of systemic involvement and the timely management of potential complications associated with both conditions.

## Discussion

HUV is a rare small-vessel vasculitis (7), first described and individualized in 1973 by McDuffie et al. (1), with an estimated incidence of 0.5 cases per 100,000 individuals (3). It typically affects females, with a mean age at diagnosis of 45 years (4). Idiopathic HUV is defined by two major criteria: chronic urticarial lesions and hypocomplementemia, along with at least two minor features such as leukocytoclastic vasculitis, arthralgia or arthritis, ocular inflammation, glomerulonephritis, abdominal pain, or anti-C1q antibodies (8). Historically, its diagnosis excluded conditions such as systemic lupus erythematosus (SLE), cryoglobulinemia, and hereditary complement deficiencies. However, increasing overlap between HUV and connective tissue diseases—particularly SLE—has been documented. HUV is reported in approximately 7–8% of SLE patients, and up to 54% of HUV patients may develop SLE during follow-up (5, 9). Although the clinical manifestations are similar, they primarily differ in frequency (2, 9) (**Supplementary Table S1**).

### The HUV–SLE overlap conundrum

In the present case, the clinical and immunologic profile showed significant overlap between the two entities, complicating diagnostic classification. This reflects the ongoing debate in the literature as to whether HUV represents a distinct entity, a precursor, or part of the broader SLE spectrum. Although a female predominance is consistently reported in the literature (2), the fact that our patient is male adds to the rarity of this presentation and underscores the importance of recognizing atypical cases.

Cutaneous involvement in HUV is nearly constant and includes persistent urticarial papules, often with post-inflammatory pigmentation (10). These lesions are commonly associated with pain or burning sensations, but may also present with pruritus, as observed in our patient (2). Leukocytoclastic vasculitis is typical, in contrast to the non-vasculitic urticaria observed in a minority of SLE patients (9). Angioedema—another hallmark of HUV—occurs in over 50% of cases, compared to fewer than 5% in SLE (2, 9). Our patient presented with both urticarial vasculitis and angioedema.

Articular symptoms, ocular inflammation (episcleritis), and gastrointestinal manifestations are shared features of both HUV and SLE, though their frequency and patterns differ (2, 9). In this case, the spontaneous resolution of diffuse intestinal wall thickening argues against lupus enteritis and supports an HUV-related gastrointestinal involvement (6, 11). The presence of episcleritis—more common in HUV than in SLE—further supports this association. Cardiac and renal manifestations were notably absent, which helped guide a conservative therapeutic strategy.

HUV is classically associated with immune complex–mediated vascular injury and the presence of anti-C1q antibodies (6). Experimental data suggest that C1q-opsonized immune complexes may promote immune dysregulation by activating human T cells to secrete TNF-α and IFN-γ (12), though the role of T cells in HUV pathogenesis remains unclear. While anti-C1q antibodies are also found in SLE, they differ in binding properties and are more consistently elevated in HUV (90–100%) (13). Notably, unlike in SLE, about 60% of anti-C1q antibodies in HUV can bind to reduced or denatured C1q (2, 5, 14). Their presence—particularly in association with hypocomplementemia and cutaneous vasculitis—may support a diagnosis of HUV or an overlap syndrome. Anti-C1q antibodies appear to be associated with a specific clinical phenotype, characterized by a higher frequency of angioedema, livedo, ocular, articular, and renal involvement, and a lower frequency of pulmonary and gastrointestinal manifestations (15). Unlike our patient, most HUV cases (90–100%) show persistently low C1q levels, compared to 20–50% in SLE.

ANA are found in 61–71% of HUV cases, while anti-DNA and anti-SSA/SSB antibodies are each present in about 17% (9). Their detection—while not specific—may reflect an underlying systemic autoimmune process and should prompt consideration of SLE, HUV, or an overlap syndrome, particularly when accompanied by compatible clinical and immunological findings.

Some authors have proposed classification systems distinguishing primary HUV, HUV syndrome (HUVS), and secondary HUV associated with autoimmune diseases (11). However, a consensus remains lacking, and rare cases such as ours illustrate the clinical complexity of HUV–SLE coexistence. While some consider HUV a precursor to SLE, others view the two conditions as partially overlapping entities with shared immunological mechanisms.

In our case, current evidence does not allow a definitive conclusion as to whether this represents an overlap syndrome or a secondary manifestation of SLE. This ambiguity mirrors the broader uncertainty in the literature. **Table 1** summarizes the clinical and immunological features observed in our case compared to those previously reported (3, 9, 16).

**Table 1:** Clinical-immunological profile of HUV associated with SLE: Case and literature review (3, 9, 16).

Parameters	Our Case	Aydogan K et al. (9)	Tracy DeAmicis (16)	Vartika et al. (3)
Clinical Manifestations				
Age (years)	34	55	12	60
Gender	M	F	F	M
Urticaria	+	+	+	+
Leukocytoclastic vasculitis	+	+	+	+
Angioedema	–	+	+	+
Fever	–	+	+	–
Leukopenia	+	+	–	–
Thrombocytopenia	–	–	–	–
Auto-immune haemolysis	–	–	–	–
Delirium/Psychosis/Seizure	–	–	–	–
Non-scarring alopecia	–	–	+	–
Oral ulceration	–	–	–	–
Cutaneous lupus lesion	–	+	–	–
Arthralgia/Arthritis	+	+	+	+
Ocular involvement (Scleritis/Episcleritis)	Episcleritis	Episcleritis	–	–
Abdominal pain	+	–	+	–
Renal involvement	–	–	+	+
Obstructive syndrome	–	–	–	+
Restrictive syndrome	–	–	–	–
Pleuro-pericardial effusion	–	–	–	–
Pericarditis	–	–	–	–
Immunological Findings				
Anti-nuclear antibodies	+	+	+	+
C1q	N	NF	NF	NF
C3	↘	↘	↘	↘
C4	↘	↘	↘	↘
Anti-C1q antibodies	+	NF	NF	+
Anti-DNA antibodies	+	–	–	–
Anti-Sm antibodies	+	+	+	–
Anti-SSA antibodies	+	+	–	–
Anti-β2-glycoprotein I antibodies Anticardiolipin antibodies Lupus anticoagulant	–	–	+	–
ANCA	–	–	NF	–

M, Male; F, Female; NF, Not Found; N, Normal; ↘, Low; +, Positive; -, Negative.

In our patient, the therapeutic approach was guided by a relatively mild clinical presentation, the feasibility of close monitoring, and the well-established immunomodulatory effects of hydroxychloroquine (HCQ) in both SLE and vasculitic conditions. Notably, the absence of major organ involvement—particularly renal and pulmonary—contrasts with the literature, where such features are reported in approximately 50–65% of overlap cases (9, 15). The patient expressed satisfaction with the rapid improvement in symptoms and adherence to treatment was good.

This atypical presentation highlights the clinical heterogeneity of HUV–SLE overlap and directly informed our decision to initiate HCQ monotherapy. The patient responded favorably, with no requirement for corticosteroids or additional immunosuppressive agents. This suggests that, in selected cases with mild-to-moderate disease and no visceral involvement, a conservative treatment strategy may be sufficient.

Nevertheless, this approach may not be generalizable, and close clinical monitoring remains essential. The therapeutic management of HUV is not standardized, with limited evidence-based guidelines available. Antihistamines—even at high doses—are consistently ineffective for both cutaneous and systemic symptoms. Treatment often parallels that of SLE, with agents such as colchicine, HCQ, and dapsone frequently used in mild cases, although their efficacy can vary (15).

Diagnosing HUV is particularly challenging due to its rarity and its broad spectrum of clinical manifestations. When it coexists with SLE, prognosis tends to be poorer due to increased systemic involvement and greater therapeutic complexity (9). Although rare, the coexistence of HUV and SLE poses significant diagnostic and therapeutic challenges. This case adds to the limited literature on this topic and emphasizes the utility of anti-C1q testing in SLE patients presenting with urticarial vasculitis.

## Conclusion

This case underscores the diagnostic and therapeutic challenges associated with the rare overlap between Hypocomplementemic urticarial vasculitis (HUV) and systemic lupus erythematosus (SLE). These two conditions share key clinical, immunological, and pathophysiological features, suggesting they may exist along a continuum within the spectrum of autoimmune diseases. In our patient, the absence of major organ involvement and the favorable response to hydroxychloroquine monotherapy highlight the clinical heterogeneity of HUV-SLE overlap and support the potential for a conservative treatment approach in selected cases with mild-to-moderate disease. Nevertheless, due to the rarity and variable presentation of this overlap, individualized management remains essential. Further research is needed to better delineate the disease spectrum and to establish clear, evidence-based therapeutic guidelines for managing overlap syndromes.

## Data Availability

The original contributions presented in the study are included in the article/**Supplementary Material**. Further inquiries can be directed to the corresponding author.
