# Supplementary material for: Urticarial hypocomplementemic vasculitis syndrome and systemic lupus erythematosus: a case report and review of the literature
Source: Front Immunol. 2025 Aug 7;16:1649699. doi: 10.3389/fimmu.2025.1649699 (PMC12368617; doi:10.3389/fimmu.2025.1649699)
Supplement: Supplementary file 1 [file DataSheet1.pdf]

Suppl table 1. Frequency of clinical manifestations in HUV and SLE in the literature (2,9)

Clinical manifestation	HUV (%)	SLE (%)
Urticaria with leukocytoclastic vasculitis	100	<10
Angioedema	72	<5
Arthralgia and/or arthritis	100	95
Obstructive pulmonary involvement	65	24-30
Ocular involvement	61	15
Renal involvement	50	36-50
Pericarditis	17	30
